# The Multifaceted Roles of Pyroptotic Cell Death Pathways in Cancer

**DOI:** 10.3390/cancers11091313

**Published:** 2019-09-05

**Authors:** Man Wang, Shuai Jiang, Yinfeng Zhang, Peifeng Li, Kun Wang

**Affiliations:** 1 Institute for Translational Medicine, Medical College of Qingdao University, Dengzhou Road 38, Qingdao 266021, China; zhangyinfeng@qdu.edu.cn (Y.Z.); peifli@qdu.edu.cn (P.L.); 2 Key Laboratory of Experimental Marine Biology, Institute of Oceanology, Chinese Academy of Sciences, Qingdao 266071, China; sjiang@qdio.ac.cn

**Keywords:** pyroptosis, cancer, inflammasome, gasdermin, pro-inflammatory cytokine, cancer pathogenesis, therapeutic utility

## Abstract

Cancer is a category of diseases involving abnormal cell growth with the potential to invade other parts of the body. Chemotherapy is the most widely used first-line treatment for multiple forms of cancer. Chemotherapeutic agents act via targeting the cellular apoptotic pathway. However, cancer cells usually acquire chemoresistance, leading to poor outcomes in cancer patients. For that reason, it is imperative to discover other cell death pathways for improved cancer intervention. Pyroptosis is a new form of programmed cell death that commonly occurs upon pathogen invasion. Pyroptosis is marked by cell swelling and plasma membrane rupture, which results in the release of cytosolic contents into the extracellular space. Currently, pyroptosis is proposed to be an alternative mode of cell death in cancer treatment. Accumulating evidence shows that the key components of pyroptotic cell death pathways, including inflammasomes, gasdermins and pro-inflammatory cytokines, are involved in the initiation and progression of cancer. Interfering with pyroptotic cell death pathways may represent a promising therapeutic option for cancer management. In this review, we describe the current knowledge regarding the biological significance of pyroptotic cell death pathways in cancer pathogenesis and also discuss their potential therapeutic utility.

## 1. Introduction

Cell death is a crucial phenomenon in biological activities and serves a pivotal role in maintaining homeostatic balance in vivo [1]. At present, several forms of cell death, such as apoptosis, necroptosis and pyroptosis, have been found [2]. As a form of non-inflammatory programmed cell death, apoptosis can be induced by either intrinsic or extrinsic factors, followed by sequential activation of initiator and executioner caspases [3,4]. Both apoptosis and pyroptosis are executed by caspases. Apoptosis is mediated by caspase-2, -3, -6, -7, -8 and -9 [5]. The term pyroptosis was initially proposed in 2001. Pyroptosis is inherently a type of programmed cell death that is initiated by inflammatory caspases (caspase-1, -4, -5 and -11) upon activation of the canonical or non-canonical inflammasome pathways [6]. The requirement of inflammatory caspases in executing pyroptosis differentiates it from another inflammatory and necrotic form of programmed cell death known as necroptosis [7]. Necroptosis is a form of regulated cell death mediated by receptor-interacting serine/threonine-protein kinase 3 (RIPK3) (all the abbreviations are listed in Table S1) and its downstream substrate mixed lineage kinase domain-like pseudokinase (MLKL) [8]. RIPK3 phosphorylates the necroptosis executioner MLKL, resulting in the formation of MLKL oligomers, which then translocate to the plasma membrane [9]. These events ultimately lead to necrotic plasma membrane permeabilization and cell death associated with loss of cell and organelle integrity [10]. Necroptosis is widely known as a defense mechanism against viral infection and can induce programmed cell death in virus-infected cells.

Recently, pyroptosis has become a research hotspot in programmed cell death. The induction of pyroptosis requires the activation of the pore-forming protein gasdermin D (GSDMD) by inflammatory caspases [11]. In the canonical inflammasome pathway, caspase-1 mediates the cleavage of GSDMD and the maturation of pro-inflammatory cytokines, interleukin-1β (IL-1β) and interleukin-18 (IL-18) [12]. GSDMD pores favor the leakage of intracellular components into the extracellular environment. Unlike canonical inflammasomes, the non-canonical inflammasome pathway can be initiated by the direct binding of caspase-4, -5 and -11 to lipopolysaccharide (LPS) from Gram-negative bacteria [13]. These caspases activate GSDMD to induce cell lysis and death. In addition, caspase-1 is activated in the non-classical pyroptosis pathway, leading to the production of IL-1β and IL-18, which are liberated into the extracellular milieu [14]. Pyroptosis serves a vital role in host defense response against pathogens [15]. Pyroptotic cell death, started by pathogen infection, contributes to the release of cytosolic contents from infected cells, hence triggering an inflammatory cascade [16]. Local inflammation results in recruitment and activation of immune cells, ultimately facilitating the clearance of invading pathogens. Of note, recent studies have confirmed that pyroptosis functions in orchestrating cancer cell death [17,18]. Moreover, inflammasomes, gasdermins and pro-inflammatory cytokines can regulate key processes involved in cancer development. Therefore, pyroptotic cell death pathways constitute a novel mechanism contributing to cancer pathogenesis. In this review, we summarize the recent findings related to the role and mechanism of pyroptotic cell death pathways in cancer progression and discuss potential therapeutic values in targeting pyroptosis for cancer therapy.

## 2. The Characteristics of Pyroptosis

**Figure 1:**
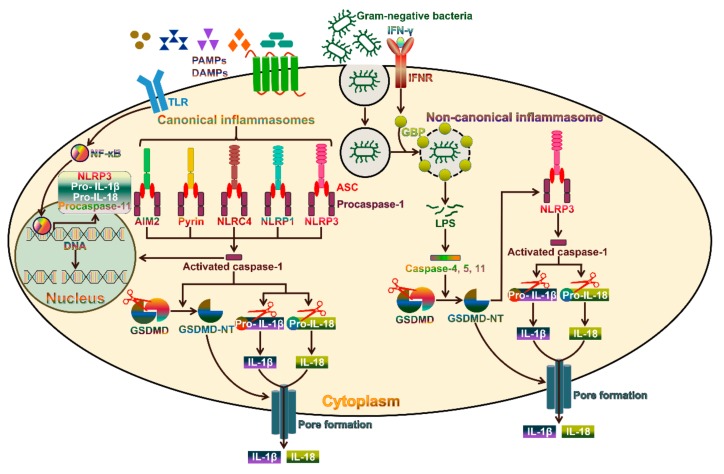
Molecular mechanisms involved in inflammasome activation. Activation of the canonical inflammasomes usually requires two steps. As the first priming step, PAMPs or DAMPs combine with TLRs on the plasma membrane to trigger NF-ĸB-dependent transcription of inflammasome components and their downstream targets, including NLRP3, pro-IL-1β and pro-IL-18. The second step involves the recognition of PAMPs or DAMPs by the inflammasome sensors AIM2, pyrin, NLRC4, NLRP1 and NLRP3. These sensors recruit the adaptor protein ASC and procaspase-1 to form diverse inflammasomes. Once assembled, the inflammasomes act as activating platforms for procaspase-1. Catalytically active caspase-1 converts the inactive pro-IL-1β and pro-IL-18 into their biologically active forms (IL-1β and IL-18). On the other hand, caspase-1 cleaves GSDMD to release an N-terminal pore-forming domain (GSDMD-NT), which migrates toward the plasma membrane and forms membrane pores. The pores facilitate the release of the active IL-1β and IL-18 into the extracellular environment. In addition, caspase-1 activation leads to the cleavage of chromosomal DNA by nuclease activity. The non-canonical inflammasome pathway is specifically activated by Gram-negative bacteria. IFN-γ-induced GBP causes the lysis of Gram-negative bacteria-containing vacuoles, allowing the release of LPS into the cytoplasm. Intracellular bacterial LPS can directly activate caspase-4 and -5 in humans and caspase-11 in mice. These caspases cleave GSDMD to drive pyroptotic cell death. GSDMD-NT activates the NLRP3 inflammasome to promote caspase-1-dependent cytokine maturation. TLR, toll-like receptor; PAMPs, pathogen-associated molecular patterns; DAMPs, damage-associated molecular patterns; IFN-γ, interferon-γ; IFNR, interferon receptor; NF-ĸB, nuclear factor-ĸB; GBP, guanylate-binding protein; ASC, apoptosis-associated speck-like protein containing a caspase recruitment domain; LPS, lipopolysaccharide; GSDMD, gasdermin D; GSDMD-NT, the N-terminal fragment of GSDMD; IL-1β, interleukin-1β; IL-18, interleukin-18.

Pyroptosis, a pro-inflammatory form of cell death, is generally induced by intracellular pathogen infection and forms part of the host defense system [19]. Pyroptosis can be induced via two pathways, the canonical and non-canonical inflammasome pathways (Figure 1). Canonical pyroptosis is executed by caspase-1, which is triggered by a number of pathogen-associated molecular patterns (PAMPs) and damage-associated molecular patterns (DAMPs), while non-canonical pyroptosis is dependent on human caspase-4/-5 or mouse caspase-11 and can be induced by intracellular LPS [20]. The morphological features of caspase-1-dependent pyroptosis and caspase-1-independent pyroptosis are similar. Both are characterized by the loss of cellular membrane integrity, chromatin condensation and DNA fragmentation. Specially, the cellular membrane undergoes rupture, resealing and swelling, and forms a balloon-shaped vesicle around the nucleus [21]. The cellular membrane becomes disrupted, and the cytoplasmic materials, including pro-inflammatory cytokines, endogenous DAMPs and alarmins, are released into the extracellular space [16].

## 3. The Canonical Pyroptosis Pathway

Inflammasomes are macromolecular protein complexes consisting of an inflammasome sensor, the adaptor protein apoptosis-associated speck-like protein containing a caspase recruitment domain (ASC) and caspase-1 [22]. Based on their structural features, the inflammasome sensors are categorized into nucleotide-binding oligomerization domain (NOD)-like receptors (NLRs), absent in melanoma 2 (AIM2)-like receptors (ALRs) and pyrin [23]. The NLRs belong to host pattern recognition receptors (PRRs) and act as intracellular sensors of PAMPs and DAMPs [24]. The NLRs are composed of three main domains: An N-terminal caspase recruitment domain (CARD) or pyrin domain, a middle nucleotide-binding domain (NBD or NACHT) and a C-terminal leucine-rich repeat (LRR) domain [25]. The NBD domain mediates NLR oligomerization, while the LRR acts as a sensor of PAMPs and DAMPs [26]. Once formed, the N-terminal pyrin or CARD domain of NLR oligomers recruits and seeds ASC or caspase-1. Among NLRs, NLR family pyrin domain-containing 1 (NLRP1), NLRP2, NLRP3, NLRP6, NLRP7, NLRP12 and NLR family CARD domain-containing protein 4 (NLRC4) are regarded as inflammasome-nucleating proteins [27]. The ALRs are a newly characterized class of PRRs that can detect pathogen DNAs in both the cytosol and nucleus [28]. The ALR family member AIM2 consists of an N-terminal pyrin domain, which binds to the pyrin domain of ASC, and a C-terminal hematopoietic interferon (IFN)-inducible nuclear protein containing a 200-amino-acid repeat (HIN-200) domain, which directly interacts with dsDNA [29]. AIM2 can initiate inflammasome assembly upon activation leading to caspase-1-mediated inflammatory responses and cell death [30]. Pyrin is another vital inflammasome-forming protein [31]. Pyrin harbors an N-terminal pyrin domain that is responsible for its combination with ASC and subsequent activation of caspase-1 [32].

Five canonical inflammasomes, NLRP1, NLRP3, NLRC4, AIM2 and pyrin, have been identified [33]. These inflammasomes can be triggered by different stimuli. For instance, the mouse NLRP1b and rat NLRP1 inflammasome sensors can be activated after their cleavage by a lethal factor released by the Gram-positive bacterium *Bacillus anthracis* [34,35]. NLRP3 mainly recognizes viral dsRNAs, bacterial toxins, reactive oxygen species (ROS) and endogenous damage signals [32]. NLRC4 responds to bacterial protein stimulation, while AIM2 is predominantly responsible for the recognition of cytoplasmic dsDNAs during bacterial or viral infection [36,37]. Pyrin is activated by bacterial toxins that modify RhoA GTPases [38]. The adaptor protein ASC bridges the interaction between the sensor protein and procaspase-1 within the canonical inflammasome [39]. ASC recruits procaspase-1 via a CARD–CARD domain interaction [40]. Remarkably, ASC is indispensable for the pyrin domain-containing sensors (NLRP3, AIM2 and pyrin) to recruit procaspase-1, while the CARD-based sensors (NLRP1b and NLRC4) can directly bind to procaspase-1 [32]. After being recruited to the inflammasome, procaspase-1 forms dimers and activates its own protease capability to generate caspase-1 [15]. Caspase-1-mediated cell death represents the canonical pyroptosis pathway. Activated caspase-1 induces the proteolytic processing of the pro-inflammatory precursor cytokines (pro-IL-1β and pro-IL-18) to release active IL-1β and IL-18 [41]. The pro-pyroptotic factor GSDMD consists of an N-terminal pore-forming domain and a C-terminal repressor domain (RD). The RD domain binds the GSDMD-NT and maintains the protein in an autoinhibitory state [42]. Caspase-1 activated by the canonical inflammasomes induces the cleavage of GSDMD, liberating the N-terminal fragment (GSDMD-NT) [11]. In the canonical pyroptosis pathway, the formation of inflammasomes is required for caspase-1-mediated cleavage of GSDMD. Caspase-1, -4, -5 and -11 cleave GSDMD at an aspartate residue in the linker that connects GSDMD-NT and RD, which leads to the generation of a noncovalent GSDMD-NT-RD complex [43]. Intriguingly, GSDMD-NT has high affinity for specific lipid compositions, such as phosphatidic acid, phosphatidylserine, cardiolipin, mono- and bisphosphorylated phosphoinositols [44]. As phosphatidylserine and phosphoinositols are restricted to the inner leaflet of the plasma membrane, GSDMD-NT can only oligomerize to form pores from the cytosolic face [45]. Upon lipid binding, the N-terminal domain of gasdermin A3 (GSDMA3) underwent significant conformational changes, leading to its separation from the RD domain and oligomerization into a ring-shaped structure [46]. In addition, the conformational changes also facilitated membrane insertion of the ring architecture. Considering the similar structural and biochemical features between GSDMD and GSDMA3, this mechanism could apply to the formation of GSDMD-NT pores. Moreover, cleaved GSDMD exhibits no affinity for the outer leaflet of the cellular membrane, avoiding damage to surrounding cells during pyroptotic cell death [44]. GSDMD-NT-formed pores mediate osmotic cell swelling, plasma membrane rupture and the liberation of intracellular components including IL-1β and IL-18 [47]. Additionally, caspase-1 plays an important role in triggering DNA fragmentation.

GSDMD-NT pores act as the conduit for potassium (K^+^) efflux that sufficiently triggers the activation of the NLRP3 inflammasome [48,49]. Caspase-11 could activate the canonical NLRP3 inflammasome by boosting GSDMD-induced K^+^ efflux, demonstrating that canonical and non-canonical inflammasomes functioned synergistically to protect the host against pathogen invasion [50]. The influx of calcium (Ca^2+^) ions from the extracellular environment also occurs through GSDMD-NT-induced pores [6]. Interestingly, GSDMD-NT pores did not necessarily lead to cell death, since Ca^2+^ influx served as a signal for cells to initiate membrane repair program. Moreover, the repair mechanism involved recruitment of the endosomal sorting complexes required for transport (ESCRT) machinery to damaged membrane sites. Accordingly, suppression of the ESCRT-III machinery significantly promoted pyroptotic cell death downstream of GSDMD activation. In the pyroptosis pathway, the GSDMD-NT pore serves as a channel for release of IL-1β and IL-18. Notably, these inflammatory cytokines can be released by alternative mechanisms. For instance, activated caspase-1, pro-IL-1β and pro-IL-18 can be encapsulated into secretory lysosomes [51]. Caspase-1 processes pro-IL-1β and pro-IL-18 to generate bioactive cytokines within secretory lysosomes. The mature cytokines are then released into the extracellular milieu via fusion of lysosomes with the plasma membrane. Moreover, caspase-1-mediated IL-1β cleavage triggered its translocation from the cytosol to plasma membrane and was sufficient for GSDMD-independent IL-1β release [52]. In contrast, caspase-1 and GSDMD could accelerate IL-1β secretion. During necroptosis, MLKL activation induced the assembly of the NLRP3 inflammasome and caused plasma membrane rupture [53]. These events resulted in the maturation and release of IL-1β. Thus, IL-1β and IL-18 can be released into the extracellular space through GSDMD-independent mechanisms.

## 4. The Non-Canonical Pyroptosis Pathway

In addition to the above-described canonical inflammasome pathway, the non-canonical inflammasome was identified [54]. The molecular mechanisms underlying the actions of the non-canonical inflammasome are still being defined. It has been reported that human caspase-4 and -5, the orthologues of mouse caspase-11, serve as receptors for intracellular LPS [13]. Unlike caspase-1, caspase-4, -5 and -11 do not require an upstream signaling pathway to sense LPS, but directly detect LPS [13]. Notably, caspase-11 cannot be activated unless LPS gains access to the cytosol. Specifically, caspase-4, -5 and -11 directly bind to the lipid A moiety of LPS from Gram-negative bacteria in the cytoplasm via their CARD domains. The binding of LPS to caspase-4, -5 and -11 promotes their oligomerization and activation. These activated caspases then cleave GSDMD to generate biologically active GSDMD-NT, contributing to pyroptotic cell death. Thus, in the non-canonical pyroptosis pathway, inflammasome assembly is not necessary for caspase-4, -5 and -11-mediated cleavage of GSDMD. GSDMD-NT causes the activation of caspase-1-dependent NLRP3 inflammasome, resulting in the secretion of IL-1β and IL-18 [55]. Recently, the apoptotic caspase-8 was found to cleave GSDMD, leading to pyroptotic cell death in murine macrophages [56]. However, caspase-8 was less efficient at mediating GSDMD cleavage than caspase-1. Downstream of caspase-8-mediated GSDMD cleavage, the NLRP3 inflammasome was activated, leading to the maturation and secretion of pro-inflammatory cytokines.

## 5. The Role of Inflammasomes in Cancer

It is well-known that chronic inflammation can propel cancer occurrence. The inflammasome is a multiprotein complex that can modulate innate and adaptive immune responses. Increasing evidence demonstrates that inflammasomes are implicated in cancer growth, invasion and metastasis [57]. Several inflammasomes including NLRP3, NLRP1, NLRC4, pyrin and AIM2 play a vital role in cancer pathogenesis through their regulation of immunity and apoptosis [58]. Among these inflammasome complexes, the NLRP3 inflammasome is the best characterized and can be activated by inflammatory infections and endogenous stimuli, such as pathogens, pore-forming toxins and adenosine triphosphate (ATP) [59]. NLRP3 consists of a pyrin domain, a central NBD and an LRR region. Upon activation, NLRP3 interacts with the pyrin domain of ASC through homophilic interactions [60]. The adaptor ASC in turn recruits procaspase-1 via a CARD–CARD interaction [40].

The roles of NLRP3 in cancer occurrence and progression have been intensively studied. Polymorphisms of the NLRP3 inflammasome were associated with various malignancies including melanoma and colon cancer [61]. The effect of NLRP3 on immune regulation represents a vital mechanism mediating its pro-tumorigenic action. For instance, the NLRP3 inflammasome/IL-1β pathway enhanced immunosuppressive cell accumulation to facilitate the tumorigenesis of head and neck squamous cell carcinoma (HNSCC) [62]. Another study showed that the NLRP3 inflammasome was correlated with the carcinogenesis and development of cancer stem cells (CSCs) in HNSCC [63]. The NLRP3 inflammasome was shown to be engaged in inflammation-related lung carcinogenesis [64]. NLRP3 also promoted lung tumorigenesis induced by benzo(a)pyrene plus LPS in mice [65]. It was reported that inactivation of the NLRP3 inflammasome repressed tumor growth and immunosuppression in breast cancer [66]. Additionally, NLRP3 promoted the expansion of immune-suppressive macrophages in pancreatic ductal adenocarcinoma (PDAC), thus facilitating the generation of tumor-promoting T helper type 2 (Th2) cells and inhibiting the activation of cytotoxic CD8^+^ T cells [67]. Therefore, the NLRP3 signaling primed macrophage-induced adaptive immune suppression in PDAC. Targeting NLRP3 might be a promising immunotherapeutic strategy for PDAC.

Several literatures have shown that the NLRP3 inflammasome orchestrates cancer pathogenesis by governing cell death pathways. For instance, the NLRP3 inflammasome was involved in leptin-induced growth of breast cancer cells via promotion of cell cycle progression and suppression of cell apoptosis [68]. The NLRP3 inflammasome activation enhanced the proliferation and suppressed the apoptosis of lymphoma cells through upregulation of c-myc and B-cell lymphoma-2 (Bcl-2), and downregulation of tumor protein p53 (TP53) and Bcl-2-associated X protein (Bax) [69]. On the other hand, the NLRP3 inflammasome might suppress cancer progression. 17β-estradiol (E2)-induced activation of the NLRP3 inflammasome triggered pyroptotic cell death and suppressed protective autophagy in hepatocellular carcinoma (HCC) cells [70].

Epithelial-mesenchymal transition (EMT) is a well-orchestrated process by which epithelial cells can convert into cells of the mesenchymal phenotype [71]. It is generally accepted that EMT plays a vital role in tumor invasion and metastasis [72]. The NLRP3 inflammasome controls the EMT program during cancer pathogenesis. A previous study demonstrated that the NLRP3 inflammasome enhanced the migratory and metastatic abilities of colorectal cancer (CRC) cells [73]. In terms of mechanism, NLRP3 promoted the EMT process in CRC cells through modulation of Snail1 [74]. NLRP3 deletion limited the proliferation and EMT-induced invasion of pancreatic cancer (PC) cells [75]. NLRP3 enhanced the proliferation, invasion and migration of oral squamous cell carcinoma (OSCC) cells by modifying the expression of E-cadherin, vimentin and N-cadherin [76]. NLRP3 promoted the proliferation, invasion and migration, as well as suppressed the apoptosis of glioma cells [77]. Mechanistically, NLRP3 regulated glioma progression and metastasis via its effects on the EMT program and the phosphatase and tensin homolog (PTEN)/protein kinase B (Akt) signaling pathway. Activation of the NLRP3 inflammasome antagonized the suppressive effect of polydatin on the proliferation and migration of non-small cell lung cancer (NSCLC) cells [78]. Further mechanistic study indicated that the NLRP3 inflammasome ascended the expression of Snail and lowered the expression of E-cadherin in lung cancer cells [79]. Activation of the NLRP3 inflammasome promoted the metastasis of breast cancer cells [80]. It can be speculated that inactivation of the NLRP3 inflammasome leads to the blockage of cancer progression. As expected, β-hydroxybutyrate inhibited the migration of C6 glioma cells by suppressing the activation of the NLRP3 inflammasome [81]. Consequently, the NLRP3 inflammasome can modify the malignant behaviors of cancer cells.

The NLRP1 inflammasome was the first member of the NLRP family to be discovered and consists of NLRP1, the adaptor protein ASC and caspase-1 [82]. The polymorphism or mutation of the NLRP1 gene was found to be tightly associated with the progression of melanoma [83,84]. NLRP1 facilitated melanoma growth via depressing the apoptotic pathway by inhibiting the activities of caspase-2, -3, -7 and -9 [85]. NLRC4 comprises an N-terminal CARD domain, a central NBD and a C-terminal LRR domain. NLRC4 directly recruits procaspase-1 and is activated by a wide range of bacterial pathogens [86]. NLRC4 plays an important role in limiting cancer progression. NLRC4 and caspase-1 restricted CRC tumorigenesis through regulation of colonic epithelial cell proliferation and death [87]. Pyrin is composed of a pyrin domain, two B-boxes, a coiled-coil domain and a SPRY domain [88]. Pyrin associates with ASC via pyrin–pyrin domain homophilic interactions, which in turn recruits caspase-1 to form an inflammasome complex [89]. The pyrin inflammasome played a crucial role in enhancing intestinal barrier integrity and preventing colonic inflammation to restrict colon tumorigenesis [90].

AIM2 belongs to the pyrin and HIN domain-containing protein (PYHIN) family and can be activated by cytosolic dsDNAs of bacterial or viral origin [91]. The interaction between AIM2 and dsDNA results in AIM2 oligomerization, ultimately leading to inflammasome assembly. Apart from its role in host immune defense, AIM2 also acts as an oncogene in cancer. AIM2 was deregulated in several types of cancer and might represent a potential therapeutic target for cancer [92]. The AIM2 inflammasome boosted liver inflammation and proliferative responses during chemical-induced hepatocarcinogenesis [93]. AIM2 promoted the proliferation of NSCLC cells by upregulating cell cycle-related proteins cyclin B1 and cell division cycle 2 (CDC2) [94]. Consistently, inactivation of the AIM2 inflammasome by luteolin caused decreased expression of p-CDC2 (Tyr15), p21 and cyclin B1, thus inducing cell cycle arrest in NSCLC [95]. AIM2 depletion also restrained the EMT process and cell invasion in NSCLC through downregulation of vimentin and matrix metalloproteinase 9 (MMP9). Similarly, AIM2 could enhance the growth and invasion of cutaneous squamous cell carcinoma (cSCC) cells by upregulating MMP1 and MMP13 [96]. Paradoxically, AIM2 is regarded as a tumor suppressor in various cancers. For instance, AIM2 was capable of inhibiting the proliferation, invasion and migration of renal cell carcinoma (RCC) cells by upregulating autophagy-related genes (Bcl-2, Beclin-1, LC3-II and ATG5) [97]. AIM2 enhanced the apoptosis of CRC cells by inhibiting the phosphoinositide 3-kinase (PI3K)/Akt pathway [98]. AIM2 depressed the proliferation and invasion of HCC cells by blocking the mammalian target of the rapamycin (mTOR)/S6 kinase 1 (S6K1) pathway [99]. The depletion of AIM2 activated the EMT process in HCC cells through regulation of E-cadherin, vimentin and N-cadherin, thereby promoting HCC cell migration and metastasis [100]. The nanoparticles containing AIM2 could inhibit RCC growth and might offer a potential therapeutic approach for RCC treatment [101].

The inflammasome adaptor ASC affects cancer progression by interfering with the apoptotic signaling pathway in cancer cells. ASC was shown to restrain the apoptosis of gastric cancer (GC) cells through an IL-18-mediated inflammation-independent mechanism [102]. As a result, depletion of IL-18 resulted in the suppression of ASC-regulated gastric tumorigenesis. ASC reduced the growth of schwannoma cells by activating caspase-3 and -9 and upregulating Bcl-2 homology domain 3 (BH3)-interacting domain death agonist (BID) [103]. ASC negatively modulated the tumorigenesis of fibrosarcoma by activating caspase-9 and inhibiting the nuclear factor-ĸB (NF-ĸB)-related X-linked inhibitor of apoptosis protein (XIAP) [104]. ASC inhibited the proliferation, motility and invasion of lung cancer cells through downregulation of Bcl-2 and phospho-Src [105]. It has been reported that the apoptosis sponsor caspase-8 also functions as an enhancer of cancer cell migration by phosphorylation via Src [106]. ASC could combine with caspase-8 to impede its accessibility to Src, hence repressing the metastasis of melanoma cells [107]. It is well established that loss of E-cadherin expression, a hallmark of EMT, is a pivotal step impelling metastatic dissemination in multiple cancers [108]. Mechanistically, E-cadherin downregulation contributes to tumor metastasis by inducing transcription factors such as Twist [109]. ASC enhanced the invasion, migration and metastasis of OSCC cells by reducing the expression of E-cadherin [110]. The molecular mechanisms behind the effects of ASC on tumor metastasis remain to be further explored. The biological function of inflammasome components in multiple types of cancer highlights their therapeutic potential as molecular targets for cancer.

**Figure 2:**
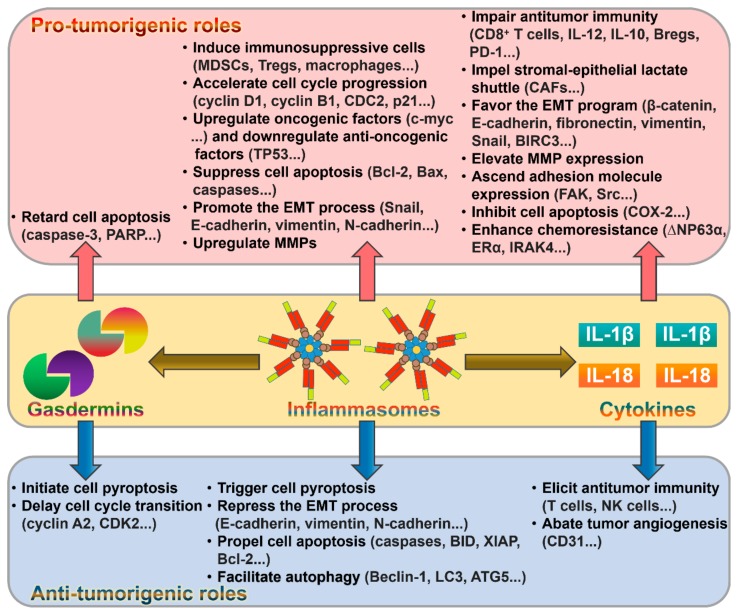
Overview of the roles of the pyroptotic pathways in cancer. The major components (inflammasomes, gasdermins and inflammatory cytokines) involved in pyroptotic cell death pathways can either promote or restrain cancer progression. Their pro-tumorigenic mechanisms primarily include induction of immunosuppressive cells, impairment of antitumor immunity, suppression of cell apoptosis, and promotion of the EMT program. Promotion of cell death, repression of the EMT process and induction of antitumor immunity represent pivotal mechanisms mediating the anti-tumorigenic roles of the pyroptotic pathways. PARP, poly (ADP-ribose) polymerase; MDSCs, myeloid-derived suppressor cells; Tregs, regulatory T cells; CDC2, cell division cycle 2; TP53, tumor protein p53; Bcl-2, B-cell lymphoma-2; Bax, Bcl-2-associated X protein; EMT, epithelial-mesenchymal transition; MMPs, matrix metalloproteinases; IL-12, interleukin-12; IL-10, interleukin-10; Bregs, regulatory B cells; PD-1, programmed cell death-1; CAFs, cancer-associated fibroblasts; BIRC3, baculoviral inhibitor of apoptosis repeat-containing 3; FAK, focal adhesion kinase; COX-2, cyclooxygenase-2; ∆NP63α, tumor protein 63 (TP63) isoform; ERα, estrogen receptor α; IRAK4, interleukin-1 receptor-associated kinase 4; CDK2, cyclin-dependent kinase 2; BID, Bcl-2 homology domain 3 (BH3)-interacting domain death agonist; XIAP, X-linked inhibitor of apoptosis protein; LC3, microtubule-associated protein light chain 3; ATG5, autophagy-related gene 5; NK cells, natural killer cells.

Although there are some studies that support the suppressive effects of inflammasome components against cancer, some reports still indicate the pro-tumorigenic functions of inflammasome constituents (Figure 2). The dual roles of the inflammasome pathways may depend on the type of tumors or tissues. Additional exploration is warranted to fully disclose the function of these multiprotein complexes in cancer pathogenesis before targeting them for therapeutic intervention in cancer. The signaling pathways or molecules initiating inflammasome activation during cancer progression should be identified. The detailed mechanisms underlying the involvement of inflammasomes in cancer pathology remain to be studied. Targeting inflammasome components or signaling pathways may provide a new opportunity for development of effective anticancer therapeutics.

Small molecules targeting inflammasome components (e.g., monoclonal antibodies and antagonists) are being developed for use in cancer management [111]. The P2X7 receptor is crucial for the activation of the NLRP3/IL-1 pathway [112]. Avastin (bevacizumab) is a recombinant humanized monoclonal antibody that can suppress angiogenesis by targeting vascular endothelial growth factor A (VEGFA) [113]. It has been approved by the U.S. Food and Drug Administration (FDA) for the treatment of various cancers [114]. Bevacizumab was shown to repress the growth and neoangiogenesis of P2X7-expressing tumors [115]. Accordingly, bevacizumab may be a potent inhibitor of the NLRP3/IL-1 pathway. Moreover, glyburide, also known as glibenclamide, was able to prevent NLRP3 activation and IL-1β production induced by microbial ligands and DAMPs [116]. Of note, accumulating evidence has proven the anticancer property of glibenclamide [117,118,119]. In addition, MCC950 was a specific small-molecule inhibitor of NLRP3 and AIM2 inflammasomes, which might represent a promising therapeutic approach for inflammasome-associated cancers [120]. Intriguingly, non-coding RNAs, including circRNAs and miRNAs, could regulate the activation of the NLRP3 inflammasome [121,122]. Thus, specific inhibitors of the NLRP3 inflammasome hold considerable promise as novel therapeutic agents aimed at treating cancers. Nevertheless, inflammasome-based therapies may cause deleterious side effects including the excessive inhibition of inflammasomes and the development of autoinflammatory or metabolic diseases. The limitation of inflammation-targeted drugs remains to be systematically investigated. Besides, their safety and efficacy must be validated before their clinical translation. A better understanding of the regulatory mechanism and clinical relevance of the inflammasome pathways in cancer will possibly open the way to the translation of small-molecules targeting inflammasomes in clinical cancer therapy.

## 6. The Contribution of Gasdermins to Cancer Progression

**Figure 3:**
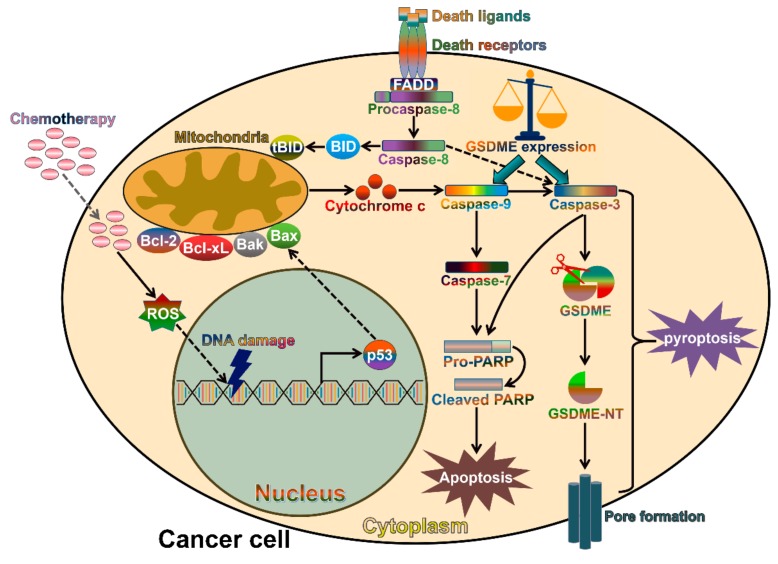
Schematic representation of chemotherapy-induced apoptosis and pyroptosis in cancer cell. Apoptosis is induced via the intrinsic or extrinsic death signaling pathway. Chemotherapy induces DNA damage and activates p53, which interacts with Bax in mitochondria and triggers the mitochondria-dependent (intrinsic) apoptotic pathway. Detailedly, Bax promotes the release of cytochrome c and initiates the capase-9/caspase-3 cascade, resulting in cell apoptosis. In contrast, the extrinsic apoptosis pathway involves the binding of death ligands (e.g., Fas and TNF) to their receptors. This event gives rise to the activation of caspase-8. The latter evokes cell apoptosis by cleaving BID and directly activating caspase-3. In certain GSDME-expressing cancer cells, GSDME is able to switch chemotherapy-induced apoptosis to pyroptosis. Caspase-3 cleaves GSDME to generate the N-terminal fragment (GSDME-NT), which perforates the plasma membrane to execute pyroptosis. Bcl-2, B-cell lymphoma-2; Bcl-xL, B-cell lymphoma-extra large; Bak, Bcl-2 homologous antagonist/killer; Bax, Bcl-2-associated X protein; BID, Bcl-2 homology domain 3 (BH3)-interacting domain death agonist; tBID, truncated BID; FADD, Fas-associated death domain protein; ROS, reactive oxygen species; PARP, poly (ADP-ribose) polymerase; GSDME, gasdermin E; GSDME-NT, the N-terminal fragment of GSDME.

Chemotherapeutic agents are capable of killing tumor cells by activating caspase-3-mediated apoptosis (Figure 3). As cancer cells usually lose their capability to initiate or execute apoptosis, they are resistant to conventional chemotherapeutic agents that target the cellular apoptosis pathway [123]. Therefore, it is necessary to identify alternative cell death pathways for effective cancer management. Notably, a recent study indicated that cleavage of gasdermin E (GSDME) by caspase-3 induced pyroptosis in neuroblastoma and melanoma cells following treatment with chemotherapeutic agents, such as DNA-binding/modifying compounds (doxorubicin, cisplatin and actinomycin-D) and the topoisomerase inhibitors (topotecan, CPT-11, etoposide and mitoxantrone) [17]. Therefore, the activation of caspase-3/GSDME-dependent pyroptosis might be an alternative therapeutic strategy for cancer treatment. Likewise, the chemotherapeutic agent cisplatin initiated the activation of caspase-3 and generation of bioactive GSDME-NT, thus eliciting pyroptosis in lung cancer cells [18]. Another study also showed that the caspase-3/GSDME pathway mediated pyroptotic cell death induced by small-molecule inhibitors in lung cancer cells [124]. Lobaplatin could induce the activation of ROS and the phosphorylation of c-Jun N-terminal kinase (JNK) in CRC cells [125]. This led to the recruitment of Bax to mitochondria and sequential release of cytochrome c, followed by caspase-3/-9 activation and GSDME-mediated pyroptosis. The anticancer agent 5-fluorouracil (5-FU) induced caspase-3-mediated cleavage of GSDME in GC cells, which switched cell apoptosis into pyroptosis [126]. These findings confirmed a vital role of GSDME-mediated pyroptotic cell death in boosting the detrimental effects of chemotherapeutic agents and provide novel insights into improved cancer therapy. Caspase-3 is activated by the apoptotic pathways. Therefore, caspase-3 does not require inflammasome platforms for its activation and subsequent cleavage of GSDME.

In humans and mice, caspase-3 specially cleaves GSDME at the tetrapeptide motif _267_DMPD_270_ [17], while caspase-1, -4, -5, -8 and -11 cleave GSDMD after the _272_FLTD_275_ sequence [42,127]. The cleavage of GSDMD and GSDME by caspases results in the liberation of the N-terminal domains (GSDMD-NT and GSDME-NT) that induce pyroptosis via their pore-forming activity. Caspases-1, -3, -4, -5, -8 and -11 are able to target multiple substrates [128]. Although each caspase possesses its preferred substrate cohort, caspase-1, -4, -5, -8 and -11 could cleave the same substrate GSDMD. The cleavage of GSDMD by caspases is triggered by different stimuli. For instance, caspase-1-mediated cleavage of GSDMD occurs in response to microbial infection or danger signals, while caspase-4, -5 and -11 are activated upon sensing of intracellular LPS to cleave GSDMD. The cleavage of GSDMD by diverse caspases enables cells to initiate pyroptosis under distinct circumstances. Caspase-8 and -3 play a crucial role in initiating cell apoptosis. Reportedly, the *Yersinia* effector YopJ served as a strong activator of caspase-8 via RIPK1 [129]. *Yersinia* infection triggered caspase-8-mediated GSDMD cleavage and subsequent pyroptotic cell death, whereas the apoptotic pathway was restrained [127]. Depletion of GSDMD caused the shift from caspase-8-dependent pyroptosis to apoptosis. Several *Yersinia* effectors were found to repress caspase-1 cleavage [130,131]. This may contribute to the restriction of caspase-1-dependent GSDMD cleavage during *Yersinia* infection. Overexpression of GSDME in cancer cells switched caspase-3-mediated apoptosis triggered by tumor necrosis factor (TNF) or chemotherapeutic agents to pyroptosis [17]. Thus, caspase activity and the abundance of its substrates may lead to the switch from apoptosis to pyroptosis. In addition, several signaling cascades, such as the type I IFN and toll-like receptor (TLR)-induced signaling pathways, might be involved in shifting from apoptosis to pyroptosis during pathogen infection [132,133]. Interestingly, caspase-3 was reported to promote apoptosis via preventing pyroptosis by inactivating GSDMD [134]. It was proposed that the apoptotic activators could dampen pyroptosis. The inhibition of GSDMD by caspase-3 may act as a negative regulatory feedback mechanism to counteract pyroptosis. Several cell death pathways may exist upon stimulation, and the pyroptotic and apoptotic processes do not act exclusively. Further studies are required to explore the complex interplay between caspase-mediated cell death pathways. Moreover, the dual roles of caspase-3 in pyroptosis await thorough investigation.

Another member of the gasdermin protein family, GSDMD, plays multifaceted roles in cancer pathogenesis. GSDMD promoted the proliferation of NSCLC cells via inhibiting apoptosis by limiting the activation of caspase-3 and poly (ADP-ribose) polymerase (PARP) [135]. However, GSDMD functions oppositely in other types of cancer. For instance, downregulation of GSDMD significantly enhanced the proliferation of GC cells by raising the expression of cell cycle-related proteins, cyclin A2 and cyclin-dependent kinase 2 (CDK2) [136]. The extracellular signal-regulated kinase (ERK), signal transducer and activator of transcription 3 (STAT3) and PI3K/Akt signaling pathways also mediated the anti-proliferative effect of GSDMD. Moreover, adeno-associated virus-mediated delivery of GSDMD caused decreased growth of schwannoma cells [137]. This strategy might be a potential therapeutic approach for clinical treatment of schwannomas.

Pyroptosis has attracted lots of attention, as it may offer potential beneficial effects on therapeutic interventions in cancer. Both GSDMD and GSDME serve an important role in executing pyroptosis. Further studies on these gasdermin family members would be fundamental to adequately dissect the molecular events underlying pyroptotic cell death in cancer. More investigations are required to disclose the precise mechanisms behind GSDMD- and GSDME-mediated pyroptotic pathways, which would provide clues for cancer chemotherapy. GSDMD shows pro-tumorigenic or anti-tumorigenic abilities in different forms of cancer. Thus, it is critical to determine the molecular mechanisms contributing to the distinct impacts of GSDMD on cancer progression. Remarkably, GSDME-mediated pyroptosis may lead to the cytotoxicity of chemotherapy in normal tissues. It is likely that blocking GSDME expression in normal tissues could attenuate the adverse effects of chemotherapeutic drugs. However, further research is warranted to support the clinical utility of GSDME-targeted therapeutics in cancer patients.

## 7. The Emerging Role of Inflammasome-Dependent Cytokines in Cancer Pathogenesis

IL-1β and IL-18 are stored as inactive proforms that reside in the cytoplasm of naive immune cells [138]. Caspase-1-mediated cleavage of pro-IL-1β and pro-IL-18 occurs during the activation of the inflammasome pathways [139]. However, aberrantly expressed IL-1β and IL-18 contribute to cancer pathology [140]. These two cytokines have emerged as pivotal regulators of tumorigenic processes that may either inhibit or promote tumor occurrence, growth, invasion and metastasis according to the tumor stage, type and microenvironment.

### 7.1. IL-1β and Cancer

The IL-1 signaling cascade is activated upon the binding of IL-1α or IL-1β to the IL-1 receptor type 1 (IL-1R1), recruiting the IL-1R accessory protein (IL-1RAcP) and the myeloid differentiation primary response protein 88 (MyD88) to the receptor complex [141]. This is followed by the phosphorylation of various kinases and the translocation of NF-ĸB to the nucleus, eventually leading to the activation of inflammatory responses [142]. IL-1 is a key mediator of innate and adaptive immune responses and plays a critical role in sensing microbial invasion and activating lymphoid cell function [143]. More importantly, the pro-inflammatory cytokine IL-1β has significant effects on tumor growth, invasiveness and metastasis. For instance, IL-1β was found to be a master cytokine in the development of breast cancer [144]. Blockage of IL-1β could induce antitumor immunity and resulted in breast cancer regression by activating CD8^+^ lymphocytes. OSCC-derived IL-1β favored stromal glycolysis and induced a lactate shuttle to cancer cells, which facilitated the proliferation of OSCC cells [145]. IL-1β enhanced the proliferation of osteosarcoma (OS) cells by modifying the NF-ĸB/miR-506/Jagged1 (JAG1) pathway [146]. Reportedly, IL-1β promoted OS cell growth through modulation of the miR-376c/transforming growth factor-α (TGFA) axis [147]. These studies implied that multiple signaling pathways mediated the pro-tumorigenic activity of IL-1β in OS cells.

IL-1β can adjust the malignant characteristics of cancer cells. IL-1β enhanced the stem-like properties of GC cells by promoting the nuclear translocation of metastasis-promoting S100 calcium-binding protein A4 (S100A4) [148]. Consistently, blockage of the IL-1β signaling repressed the EMT process in GC cells by upregulating β-catenin and E-cadherin, and downregulating fibronectin, vimentin, Snail, MMP2 and MMP9 [149]. IL-1β also favored the EMT program in CRC cells by downregulating E-cadherin and upregulating vimentin [150]. The roles of IL-1β in the invasion of breast cancer were extensively explored. The IL-1β response driven by breast cancer prevented the differentiation of metastasis-initiating cancer cells (MICs) into highly proliferative E-cadherin-positive progeny [151]. Conversely, abolishment of the pro-inflammatory response led to metastatic colonization of breast cancer. Another study showed that IL-1β induced the EMT process and promoted the malignancy of breast cancer cells by activating the IL-1β/IL-1R1/β-catenin pathway [152]. Macrophage-derived IL-1β enhanced the migration of breast cancer cells and their adhesion to lymphatic endothelial cells [153]. IL-1β enhanced the invasion of breast cancer cells via upregulating MMP-9 by activating focal adhesion kinase (FAK) and proto-oncogene tyrosine-protein kinase Src [154]. IL-1β could enhance the invasion and migration of esophageal squamous cell carcinoma (ESCC) cells by promoting EMT and inducing the NF-ĸB signaling [155]. IL-1β promoted EMT and metastasis of HCC cells by upregulating hypoxia inducible factor-1α (HIF-1α) [156]. Similarly, IL-1β induced the nuclear import of NF-ĸB as well as enhanced MMP transcription, thus promoting OSCC invasion and progression [157]. IL-1β increased the expression of fascin and promoted extracellular matrix degradation and infiltration into the collagen matrix, hence facilitating OSCC cell invasion [158]. Additionally, IL-1β could increase the expression of glutaredoxin 1 (Grx1) in OSCC cells, thus facilitating the malignant transformation process [159].

The inflammatory environment is essential for the induction of chemoresistance in cancer cells. The molecular mechanisms underlying the role of the IL-1β signaling pathway in cancer chemoresistance have been disclosed. It was found that IL-1β was able to upregulate the chemoresistance-associated gene, the tumor protein 63 (TP63) isoform ∆NP63α, contributing to the acquisition of cisplatin resistance in breast cancer cells [160]. IL-1β conferred doxorubicin resistance to breast cancer cells by elevating the expression of the baculoviral inhibitor of apoptosis repeat-containing 3 (BIRC3), known as an EMT marker [161]. Thus, IL-1β-induced chemoresistance in cancer cells might be attributed to its regulation of the EMT program. IL-1β was able to enhance tamoxifen resistance in breast cancer cells by downregulating the estrogen receptor α (ERα) [152]. Abrogation of IL-1β enabled PDAC cells to regain gemcitabine sensitivity by targeting IL-1R-associated kinase 4 (IRAK4) [162]. Altogether, these studies raise the possibility that the IL-1β signaling could be therapeutically disrupted to improve chemotherapeutic efficacy in cancer patients.

### 7.2. Therapeutic Potential of IL-1 Neutralization in Cancer

IL-1β is considered as an attractive target in cancer treatment. Three IL-1 blockers, including anakinra, canakinumab and rilonacept, have been approved [163]. The IL-1R antagonist (IL-1Ra) is a natural inhibitor of IL-1β in vivo, where it acts via occupying the IL-1R [164]. Anakinra is molecularly identical to native IL-1Ra [165]. IL-1Ra was able to block the IL-1 signaling in chronic myelogenous leukemia (CML) and inhibited the growth of leukemia stem cells (LSCs) [166]. This study provided a solid basis for further investigation of anti-IL-1 strategies to promote LSC elimination in CML. A randomized phase III trial indicated that the IL-1Ra levels had a significant association with bermekimab responsiveness in patients with advanced CRC [167]. Anakinra overcame erlotinib resistance in HNSCC xenografts but had no effect on the anticancer activity of erlotinib in HNSCC cells [168]. The effectiveness of 5-FU plus bevacizumab and anakinra was assessed in patients with metastatic colorectal cancer (mCRC) [169]. This therapeutic regimen showed good tolerance in mCRC and exhibited efficacy with long-lasting tumor stabilization. Moreover, this combination had a manageable safety profile and might be a promising treatment option for CRC patients. Anakinra plus gemcitabine attenuated the proliferation, invasion and migration of PDAC cells [170]. Combined treatment with anakinra and gemcitabine also obviously reduced the tumor burden in vivo. Therefore, anakinra in combination with gemcitabine might be an effective therapeutic approach for PDAC.

Canakinumab is a human monoclonal antibody targeting IL-1β [171]. Reportedly, anakinra or canakinumab repressed breast cancer cell metastasis, and also blocked cancer cells shed into the circulation in vivo [172]. Anakinra plus canakinumab completely controlled disease activity and inhibited neoplastic recurrence in the patient with refractory Behcet disease uveitis and concomitant bladder papillary carcinoma [173]. The therapeutic potential of canakinumab in lung cancer patients was previously investigated. A significant decline in the incidence of lung cancer was observed in patients that received 150 or 300 mg of canakinumab as compared to the placebo group [174]. Lung cancer mortality was remarkably less frequent in patients assigned to canakinumab than that in patients who received the placebo, with the effect being more prominent in the higher dose (300 mg) group [175]. Rilonacept is a soluble decoy receptor that mainly neutralizes IL-1β [176]. As expected, rilonacept could maintain inflammatory remission in patients enrolled in a clinical trial [177]. Specially, rilonacept treatment caused a rapid and sustained decrease in the severity of inflammatory syndromes [178]. Further clinical studies are demanded to assess the therapeutic effectiveness of rilonacept in cancer.

### 7.3. IL-18 and Cancer

IL-18 belongs to the IL-1 cytokine family and is constitutively expressed by most cell types including epithelial cells, fibroblasts, macrophages and natural killer (NK) cells [179]. Like IL-1β, IL-18 is activated when cleaved by caspase-1 following inflammasome activation [180]. The pro-inflammatory activity of IL-18 is tuned by its physiological inhibitor IL-18 binding protein (IL-18BP) [181]. IL-18 is involved in the carcinogenesis of multiple cancers. Accumulating evidence indicated that the IL-18 promoter genotype was correlated with the risk of PC, nasopharyngeal carcinoma (NPC), HCC and NSCLC [182,183,184,185]. Moreover, genetic polymorphisms of IL-18 were linked with the prognosis and survival of patients with acute myeloid leukemia (AML) [186]. Upregulation of IL-18 was correlated with poor overall survival in patients with multiple myeloma (MM) [187]. The mechanisms by which this pro-inflammatory cytokine controls cancer progression have been explored. IL-18 restrained the apoptosis of AML cells by elevating the expression of cyclooxygenase-2 (COX-2) [188]. IL-18 mediated estrogen-related receptor α (ERRα)-regulated proliferation and migration of CRC cells [189]. IL-18 promoted OSCC cell invasion and metastasis by reinforcing the EMT process via the Wnt/β-catenin signaling pathway [190]. These studies demonstrated that IL-18 functioned to modify the malignant behaviors of cancer cells. In addition, serum IL-18 levels were evidently lower in patients with pancreatic adenocarcinoma (PA) who had response to gemcitabine-based chemotherapy compared with chemotherapy-unresponsive patients [191]. Serum IL-18 levels might be used to predict the response to gemcitabine-based chemotherapy in PA patients.

It is well-known that IL-18 acts as a critical participant in initiating antitumor immune responses [192]. IL-18 modulates innate and adaptive immune responses through the recruitment or differentiation of immune cells, such as NK cells, T cells and monocytes [179]. Moreover, IL-18 augments IFN-γ production and cytotoxicity of NK cells, T cells and neutrophils [193]. The previous study demonstrated that IL-18 favored the IFN-γ production by CD8^+^ T cells and NK cells, thereby eliciting antitumor immunity during ESCC carcinogenesis [194]. Accordingly, IL-18 significantly depressed the proliferation and metastasis of ESCC cells [195]. Likewise, IL-18 facilitated the expansion of NK cells and altered their phenotypes in lung cancer [196]. IL-18-induced NK cells might be useful for cancer immunotherapy. Adoptive transfer of T cells engineered with a melanoma-specific T cell receptor (TCR) and inducible IL-18 resulted in enhanced antitumor T cell responses, hence suppressing melanoma growth in vivo [197]. Thus, IL-18 improved the anticancer activity of dacarbazine in malignant melanoma [198]. IL-18 served a suppressive role in HCC progression by enhancing the differentiation, activity and survival of tumor-infiltrating T cells [199]. Furthermore, IL-18 inhibited HCC growth by priming NK cells trafficked to the liver [200]. Mesenchymal stem cells-expressing IL-18 inhibited the proliferation and metastasis of breast cancer cells by activating immunocytes and immune cytokines, downregulating the proliferation marker Ki-67 and suppressing tumor angiogenesis [201]. Collectively, IL-18-based immunotherapy might be a promising therapeutic strategy for cancer.

On the contrary, IL-18 can compromise host immune responses in favor of cancer evasion. PC cell-derived IL-18 boosted the differentiation of naive B cells into regulatory B cells (Bregs) and ascended expression of programmed cell death-ligand 1 (PD-L1) in Bregs, which led to PC immune tolerance [202]. Paradoxically, IL-18 enhanced the cytotoxic activity of NK cells and T cells in PC-transplanted mice [203]. However, IL-18 enhanced the proliferation and invasion of PC cells through the NF-ĸB signaling pathway. When combined with the NF-ĸB inhibitor, IL-18 exhibited a therapeutic effect on PC. It seemed that multiple pathways simultaneously participated in IL-18-regulated PC progression. The complex interplay among these pathways might determine the final effects of IL-18 on cancer pathogenesis. Breast cancer-derived IL-18 triggered programmed cell death-1 (PD-1) expression on immunosuppressive NK cells and was associated with poor prognosis in patients with triple-negative breast cancer [204]. As expected, IL-18 was implicated in leptin-enhanced breast cancer cell invasion and migration [205]. Thus, IL-18 could drive breast cancer progression by inducing PD-1-dependent immunosuppression.

### 7.4. Potential Efficacy of Anti-IL-18 in Cancer Therapy

IL-18 shows anticancer activity in different pre-clinical models of cancer immunotherapy through the activation of NK and/or T cell responses [206]. Clinical studies have been conducted to assess the therapeutic efficacy of IL-18 in cancer patients. Ten melanoma patients and nine RCC patients were enrolled in a previous study and were assigned to different doses (100, 500, 1000 or 2000 μg/kg) of IL-18 [207]. No dose-limiting toxicity was observed. Notably, IL-18 exhibited immune regulatory activity in these patients. In a phase I clinical trial, twenty-one RCC patients, six melanoma patients and one patient with Hodgkin lymphoma were given IL-18 in doses ranging from 3 to 1000 μg/kg [208]. Only one patient administrated with 100 μg/kg IL-18 experienced transient hypotension and bradycardia during the first infusion. No other dose-limiting toxicity was observed. IL-18 administration could effectively activate immune cells (lymphocytes and monocytes). Moreover, the serum levels of IFN-γ, granulocyte-macrophage colony-stimulating factor (GM-CSF), IL-18BP and soluble Fas ligand (FasL) were elevated in these patients. Thus, IL-18 had biological effects on the immune system and might be effective in treating cancer. In a phase 2 randomized study, IL-18 was well tolerated and exhibited low toxicity in 64 patients with metastatic melanoma [209].

However, IL-18 had limited therapeutic efficacy as a single agent in cancer patients. Previously, nineteen patients with non-Hodgkin lymphoma were given rituximab in combination with IL-18 at doses of 1, 3, 10, 20, 30 and 100 μg/kg [210]. This combination elevated the expression of IFN-γ, GM-CSF, and chemokines. Moreover, objective tumor responses could be observed in five patients. IL-18 (3 μg/kg) plus pegylated liposomal doxorubicin (PLD) was safe and biologically active in sixteen patients with recurrent ovarian cancer [211]. IL-18 may be used as an immune-stimulatory molecule in combined therapy with conventional chemotherapeutic agents. Intriguingly, IL-18 plays a pro-carcinogenic role in several types of cancer. Under the condition that IL-18 is harmful, the utilization of IL-18BP to neutralize IL-18 may be a potential therapeutic approach for certain types of cancer. A previous study showed that the IL-18BP-Fc therapy restrained the lung metastasis of breast cancer cells by blocking tumor-released IL-18 [212]. IL-18BP may be an alternative treatment option for cancer patients. These promising results may propel further investigation of the clinical utility of IL-18-based therapy in cancer intervention.

## 8. Conclusions

Pyroptosis, a lytic form of programmed cell death, is widely accepted as a crucial host defense mechanism against pathogen invasion. The biological functions of the pyroptotic pathways in cancer pathogenesis have gained increasing attention in recent years. Inflammasomes, gasdermins and inflammasome-dependent cytokines play a critical role in the carcinogenesis, growth, invasion, metastasis and chemoresistance of cancer cells. The broad and complicated impacts of pyroptotic cell death pathways on cancer development are mainly attributed to their regulatory effects on apoptosis-, EMT- or immune-related signaling cascades. To systematically understand the pivotal roles of pyroptosis in cancer, numerous questions must be addressed. The complex signaling mechanisms responsible for the regulation of pyroptosis in cancer cells has yet to be fully deciphered. Therefore, a deeper investigation into this mode of cell death is urgently required, either alone or alongside other cell death processes including necrosis and apoptosis. Further studies are warranted to disclose the underlying mechanisms of pyroptosis as well as its exact functions in cancer pathogenesis. The inflammasomes and pro-inflammatory cytokines act as a double-edged sword in cancer occurrence and development. It is likely that the functions of these critical components of the pyroptotic pathways in carcinogenesis differ depending on various factors, such as cell or tissue type, and tumor stage. Therefore, it is essential to disclose the pro-carcinogenic and anti-carcinogenic mechanisms by which the pyroptotic pathways orchestrate different stages of cancer development. In addition, various inflammasomes are present in cancer cells. Further studies are required to figure out how specific inflammasomes are activated or how they interact with each other during cancer progression. Furthermore, the influences of each inflammasome on host antitumor immunity and cancer immunotherapy are awaiting elucidation.

A growing number of studies have proven the regulatory roles of inflammasome-dependent cytokines (IL-1β and IL-18) in cancer development. Both IL-1β and IL-18 possess the ability to govern the antitumor immunity. Nevertheless, the detailed mechanisms by which these cytokines affect antitumor immunity and remodel the tumor microenvironment merit further investigation. Multiple types of cell, including tumor cells and stromal cells, can produce and release IL-1β and IL-18 into the tumor microenvironment. The precise function of the two cytokines secreted by diverse cells in modifying the inflammatory milieu should be characterized in further studies. The regulatory mechanisms underlying the maturation and secretion of IL-1β and IL-18 into the tumor microenvironment require profound elucidation. It is well established that the pyroptotic pathways can drive the maturation and secretion of IL-1β and IL-18. However, the significance of pyroptosis in the production of IL-1β and IL-18 remains to be further explored. In addition, the molecular mechanisms mediating the pro- or anti-carcinogenic roles of IL-1β and IL-18 deserve systematical investigation. Conventional chemotherapeutic agents are able to trigger caspase-3-induced pyroptosis in cancer cells. The regulation of pyroptotic cell death pathways in combined with chemotherapeutic drugs would be a promising therapeutic strategy for cancer. At present, only a few literatures have addressed the activation of pyroptosis by chemotherapy. The molecular mechanisms behind the activation of pyroptosis in chemotherapy-treated cancer cells await thorough elucidation. In particular, the signaling pathways that control the transition from chemotherapy-induced apoptosis to pyroptosis still need to be fully deciphered. Additional efforts should focus on comprehensively delineating the mechanisms of action for the induction of pyroptotic cell death in cancer intervention. Both GSDMD and GSDME are responsible for executing pyroptosis via their pore-forming activity. More work is demanded to explore how GSDMD- or GSDME-mediated pyroptotic pathways are selectively activated by different stimuli. It is intriguing whether GSDMD-induced pyroptosis occurs in cancer cells. The factors accounting for the switch between the two pyroptotic pathways should also be identified. Collectively, a better understanding of the molecular mechanisms underlying pyroptotic cell death will be conducive to the development of alternative therapeutic strategies for cancer treatment.

## Supplementary Materials

The following are available online at https://www.mdpi.com/2072-6694/11/9/1313/s1, Table S1: List of abbreviations used in this review.
