# Supplementary material for: The Multifaceted Roles of Pyroptotic Cell Death Pathways in Cancer
Source: Cancers (Basel). 2019 Sep 5;11(9):1313. doi: 10.3390/cancers11091313 (PMC6770479; doi:10.3390/cancers11091313)
Supplement: Supplementary file 1 [file cancers-11-01313-s001.pdf]

Supplemental Materials

The multifaceted roles of pyroptotic cell death pathways in cancer

Man Wang, Shuai Jiang, Yinfeng Zhang, Peifeng Li and Kun Wang

Table S1. List of abbreviations used in this review.

Full name	Abbreviation
receptor-interacting serine/threonine-protein kinase 3	RIPK3
mixed lineage kinase domain-like pseudokinase	MLKL
gasdermin D	GSDMD
interleukin-1β	IL-1β
interleukin-18	IL-18
lipopolysaccharide	LPS
pathogen-associated molecular patterns	PAMPs
damage-associated molecular patterns	DAMPs
toll-like receptor	TLR
interferon-γ	IFN-γ
interferon receptor	IFNR
nuclear factor-κB	NF-κB
guanylate-binding protein	GBP
apoptosis-associated speck-like protein containing a caspase recruitment domain	ASC
the N-terminal fragment of GSDMD	GSDMD-NT
nucleotide-binding oligomerization domain	NOD
NOD-like receptors	NLRs
absent in melanoma 2	AIM2
AIM2-like receptors	ALRs
pattern recognition receptors	PRRs
caspase recruitment domain	CARD
nucleotide-binding domain	NBD/NACHT
leucine-rich repeat	LRR
NLR family pyrin domain-containing 1	NLRP1
NLR family CARD domain-containing protein 4	NLRC4
hematopoietic IFN-inducible nuclear protein containing a 200-amino-acid repeat	HIN-200
reactive oxygen species	ROS
repressor domain	RD
gasdermin A3	GSDMA3

endosomal sorting complexes required for transport	ESCRT
adenosine triphosphate	ATP
head and neck squamous cell carcinoma	HNSCC
cancer stem cells	CSCs
pancreatic ductal adenocarcinoma	PDAC
T helper type 2	Th2
B-cell lymphoma-2	Bcl-2
tumor protein p53	TP53
Bcl-2-associated X protein	Bax
hepatocellular carcinoma	HCC
epithelial-mesenchymal transition	EMT
colorectal cancer	CRC
pancreatic cancer	PC
oral squamous cell carcinoma	OSCC
phosphatase and tensin homolog	PTEN
protein kinase B	Akt
non-small cell lung cancer	NSCLC
pyrin and HIN domain-containing protein	PYHIN
cell division cycle 2	CDC2
matrix metalloproteinase 9	MMP9
cutaneous squamous cell carcinoma	cSCC
renal cell carcinoma	RCC
phosphoinositide 3-kinase	PI3K
mammalian target of rapamycin	mTOR
S6 kinase 1	S6K1
gastric cancer	GC
Bcl-2 homology domain 3 (BH3)-interacting domain death agonist	BID
X-linked inhibitor of apoptosis protein	XIAP
poly (ADP-ribose) polymerase	PARP
myeloid-derived suppressor cells	MDSCs
regulatory T cells	Tregs
interleukin-12	IL-12
interleukin-10	IL-10
regulatory B cells	Bregs
programmed cell death-1	PD-1
cancer-associated fibroblasts	CAFs
baculoviral inhibitor of apoptosis repeat-containing 3	BIRC3
focal adhesion kinase	FAK
cyclooxygenase-2	COX-2

tumor protein 63 (TP63) isoform	ΔNP63α
estrogen receptor α	ERα
interleukin-1 receptor-associated kinase 4	IRAK4
cyclin-dependent kinase 2	CDK2
microtubule-associated protein light chain 3	LC3
autophagy-related gene 5	ATG5
natural killer cells	NK cells
vascular endothelial growth factor A	VEGFA
the U.S. Food and Drug Administration	FDA
gasdermin E	GSDME
c-Jun N-terminal kinase	JNK
5-fluorouracil	5-FU
tumor necrosis factor	TNF
B-cell lymphoma-extra large	Bcl-xL
Bcl-2 homologous antagonist/killer	Bak
truncated BID	tBID
Fas-associated death domain protein	FADD
the N-terminal fragment of GSDME	GSDME-NT
extracellular signal-regulated kinase	ERK
signal transducer and activator of transcription 3	STAT3
IL-1 receptor type 1	IL-1R1
IL-1R accessory protein	IL-1RAcP
myeloid differentiation primary response protein 88	MyD88
osteosarcoma	OS
Jagged1	JAG1
transforming growth factor-α	TGFA
S100 calcium-binding protein A4	S100A4
metastasis-initiating cancer cells	MICs
esophageal squamous cell carcinoma	ESCC
hypoxia inducible factor-1α	HIF-1α
glutaredoxin 1	Grx1
IL-1R antagonist	IL-1Ra
chronic myelogenous leukemia	CML
leukemia stem cells	LSCs
metastatic colorectal cancer	mCRC
IL-18 binding protein	IL-18BP
nasopharyngeal carcinoma	NPC
acute myeloid leukemia	AML
multiple myeloma	MM

estrogen-related receptor α	ERRα
pancreatic adenocarcinoma	PA
T cell receptor	TCR
programmed cell death-ligand 1	PD-L1
granulocyte-macrophage colony-stimulating factor	GM-CSF
Fas ligand	FasL
pegylated liposomal doxorubicin	PLD
